# Social support perceived by elderly people in social vulnerability according to family functionality: a cross-sectional study

**DOI:** 10.1590/1980-220X-REEUSP-2022-0475en

**Published:** 2023-11-03

**Authors:** Ana Laura de Souza e Silva, Ana Carolina Ottaviani, Fabiana de Souza Orlandi, Keika Inouye, Marisa Silvana Zazzetta, Sofia Cristina Iost Pavarini, Ariene Angelini dos Santos-Orlandi

**Affiliations:** 1 Universidade Federal de São Carlos, Departamento de Enfermagem, Programa de Pós- Graduação em Enfermagem, São Carlos, SP, Brazil.; 2 Universidade Federal de São Carlos, Departamento de Gerontologia, São Carlos, SP, Brazil.; 3 Universidade Federal de São Carlos, Departamento de Enfermagem, São Carlos, SP, Brazil.

**Keywords:** Aged, Social Vulnerability, Social Support, Family, Family Relations, Anciano, Vulnerabilidad social, Apoyo social, Familia, Relaciones familiares, Idoso, Vulnerabilidade Social, Apoio Social, Família, Relações Familiares

## Abstract

**Objective:**

To compare the social support as perceived by elderly persons in a context of social vulnerability according to family functionality.

**Method:**

A cross-sectional study using a quantitative approach, carried out in São Carlos-SP, with 123 elderly people living in a context of high social vulnerability. The sample was divided into two groups: good family functionality and moderate/severe family dysfunction. Data was collected on sociodemographic characteristics, family functionality (Family APGAR) and social support (Medical Outcomes Study Social Support Scale). The Mann-Whitney, Chi-square and Fisher’s exact statistical tests were used.

**Results:**

There was a statistically significant difference between social support and family functionality (p < 0.05). The group with good family functionality obtained higher median social support scores: affective 100.00; material 95.00; information 90.00; emotional 90.00; positive social interaction 85.00; when compared to the group with moderate/severe family dysfunction: affective 86.67; material 87.50; information 70.00; emotional 65.00; positive social interaction 65.00.

**Conclusion:**

Elderly persons living in dysfunctional families have less perceived social support when compared to those living in families with good family functionality.

## RESUMO

**Objetivo:**

Comparar o apoio social percebido por pessoas idosas em contexto de vulnerabilidade social segundo a funcionalidade familiar.

**Método:**

Estudo transversal de abordagem quantitativa, realizado em São Carlos-SP, com 123 idosos inseridos em contexto de alta vulnerabilidade social. A amostra foi dividida em dois grupos: boa funcionalidade familiar e disfunção familiar moderada/severa. Foram coletados dados de caracterização sociodemográfica, funcionalidade familiar (APGAR de Família) e apoio social (Escala de Apoio Social do Medical Outcomes Study). Foram utilizados os testes estatísticos Mann-Whitney, Qui-quadrado e Exato de Fisher.

**Resultados:**

Houve diferença estatisticamente significante entre apoio social e funcionalidade familiar (p < 0,05). O grupo com boa funcionalidade familiar obteve maiores escores medianos de apoio social: afetivo 100,00; material 95,00; informação 90,00; emocional 90,00; interação social positiva 85,00; quando comparado ao grupo com disfunção familiar moderada/severa: afetivo 86,67; material 87,50; informação 70,00; emocional 65,00; interação social positiva 65,00.

**Conclusão:**

Pessoas idosas que vivem em famílias disfuncionais têm menos apoio social percebido quando comparadas àquelas que vivem em famílias com boa funcionalidade familiar.

## RESUMEN

**Objetivo:**

Comparar el apoyo social percibido por personas mayores en un contexto de vulnerabilidad social según la funcionalidad familiar.

**Método:**

Estudio transversal con abordaje cuantitativo, realizado en São Carlos-SP, con 123 personas mayores residentes en contexto de alta vulnerabilidad social. La muestra fue dividida en dos grupos: buena funcionalidad familiar y disfunción familiar moderada/severa. Se recogieron datos sobre características sociodemográficas, funcionalidad familiar (APGAR Familiar) y apoyo social (Escala de Apoyo Social del Medical Outcomes Study). Se utilizaron las pruebas estadísticas de Mann-Whitney, Chi-cuadrado y exacta de Fisher.

**Resultados:**

Hubo una diferencia estadísticamente significativa entre el apoyo social y la funcionalidad familiar (p<0,05). El grupo con buena funcionalidad familiar obtuvo puntuaciones medias de apoyo social más altas: afectivo 100,00; material 95,00; información 90,00; emocional 90,00; interacción social positiva 85,00; en comparación con el grupo con disfunción familiar moderada/grave: afectivo 86,67; material 87,50; información 70,00; emocional 65,00; interacción social positiva 65,00.

**Conclusión:**

Las personas mayores que viven en familias disfuncionales tienen menos apoyo social percibido, en comparación con los que viven en familias con buena funcionalidad familiar.

## INTRODUCTION

The main source of support for the elderly in Brazil has been historically the family^(1)^. As well as being the oldest source of support in history, the family is a vital element in the development of individuals, i.e. the first determinant of a person’s socialization^(2)^. Investigating family functionality is important in order to identify whether the family functions as a source of support or as a stressor. Therefore, it is necessary to look at the relationships established among its members, how they solve problems, face difficulties, crises and establish the organization of functions between them^(3)^.

The family can be defined as functional or dysfunctional. The dysfunctional family system is characterized by an inability and/or excess of care and protection, sometimes the result of a lack of respect for the autonomy of the members^(3)^, the inability to face barriers and perform their functions efficiently^(4)^. On the other hand, in a functional family, there is a fair distribution of roles and support among the members, and the members face conflicts and obstacles in a harmonious way, seeking resolution and emotional stability^(4)^, offering the support that elderly people need.

In the ageing context, scholars believe that social support is one of the most important aspects regarding the improvement of the living conditions and health of older people^(1)^. Some studies have shown the benefits that older people enjoy when their support network is well structured, such as maintaining functional capacity^(5)^, high levels of subjective well-being and quality of life, protection against frailty and cognitive improvement^(6,7)^. However, the absence of family support and poor housing conditions are related to an increased risk of disability and death^(8)^.

Social vulnerability is a reflection of the sociocultural, economic and educational contexts of individuals, faced with situations of absence or insufficient support from institutions, with difficulties in exercising their rights as citizens, and may have a reduced capacity to react to adverse situations. It is believed that the deprivation of financial, social, health, cultural and recreational resources in contexts of high social vulnerability can expose individuals to health-related damage and contribute to conflictive, stressful relationships and a lack of social support^(9)^.

Several studies in the literature looked at the relationship between family functionality and social support^(4,10,11)^. There have been studies analyzing the relationship between socially vulnerable elderly people and family functionality and other study variables, such as sleep, overload, caregivers and depression^(11–13)^, but there have hardly been any studies involving the social support perceived by this specific population: elderly people in a context of social vulnerability.

A study carried out in the interior of the state of Minas Gerais, BR, with 637 elderlies, found an association between family functionality and family arrangement. It was highlighted that those who lived alone had poor family functionality compared to those who lived with others. In addition, low social support and precarious ties between family members led to family insufficiency^(4)^.

A study carried out in Portugal described the personal social networks of 612 elderly people over 65, according to their structural, functional and relational-contextual characteristics. It found that 76% got their social support from family members. They also identified a significant association between good family functionality and perceived social support, mainly in the emotional, informational and material dimensions^(14)^.

Against this backdrop, recent studies show that there is an association between social support and family functionality. However, both the national and international literature in the last five years show a scarcity of studies on the relationship between family functionality and social support perceived by elderly people living in poverty. Bearing in mind that the health, quality of life, well-being and autonomy of older people can be compromised by a lack of social support and family dysfunction, this study aims to compare the social support perceived by older people in a context of social vulnerability according to family functionality.

## METHOD

### Study Design

This is an observational, cross-sectional study using a quantitative approach. It followed the guidelines of the Strengthening the Reporting of Observational Studies in Epidemiology (STROBE)^(15)^ carried out using data from the study “Factors associated with poor sleep quality in elderly caregivers”.

### Study Site

The study was carried out in São Carlos, SP, with elderly people receiving care at five Family Health Units (USF in the Portuguese acronym) in regions of high social vulnerability, based on the São Paulo Social Vulnerability Index (IPVS in the Portuguese acronym). The five USFs selected for this study have IPVS = 5(16).

The IPVS was proposed by the State Data Analysis System Foundation (SEADE in the Portuguese acronym) as an instrument for identifying regions with priority for government intervention. Its use in this research is justified by the fact that the purpose of the IPVS goes beyond the original, and is currently used in income transfer planning in education and health, as well as in university research. The most current version, from 2013, incorporates indicators: (a) socioeconomic – per capita household income, average income of the woman in charge of the household, percentage of households with per capita household income of up to half a minimum wage, percentage of households with per capita household income of up to a quarter of a minimum wage and percentage of literate heads of household; and (b) demographic – percentage of heads of household aged 10 to 29, percentage of heads of household aged 10 to 29, average age of heads of household and percentage of children aged 0 to 5^(16)^.

### Population and Selection Criteria

The population of the study encompassed elderly people aged 60 or over, registered at USFs located in the urban area of São Carlos, SP. The following inclusion criteria were adopted: being aged 60 or over, living in the same household as another elderly family member; and being registered at a USF located in a context of high social vulnerability (IPVS = 5). The precaution related to the second inclusion criterion - selecting elderly people who lived with elderly people, would avoid bias in relation to the family functionality variable which was the criterion for dividing the groups (Group with good family functionality and Group with moderate or severe family dysfunction). Elderly people who live alone or in families where there are no other individuals from the same generation may have different perceptions of family functionality when compared to those who live with other elderly people from the same generation - especially their spouses. In addition, living alone can interfere with the perception of social support (the dependent variable). Controlling for family arrangement was a precaution to avoid bias in this methodological design.

The exclusion criteria adopted were: difficulties with speech and/or hearing which could hinder communication when the questionnaires were administered. In order to study the entire population that met the selection criteria, the sample size was not calculated. Therefore, the results obtained should be considered exploratory rather than confirmatory.

Professionals from the teams at the five USFs were initially contacted to identify the households to be visited. A list was drawn up with the names, addresses and telephone numbers of elderly people who lived with at least one other elderly person, resulting in 168 households, i.e., 336 elderly people.

All the households were visited. However, elderly people from 49 households expressed an interest in not taking part in the survey (n = 98); elderly people from 32 homes could not be found even after three attempts by the researchers on different days and at different times (n = 64); elderly people no longer lived at 18 addresses (n = 36) and elderly people from three homes had died (n = 6). All the elderly people living in the remaining 66 households were interviewed, corresponding to 132 elderly individuals. Of these, nine were excluded for not answering the instruments relating to the variables of interest. The final sample therefore consisted of 123 elderly people.

### Data Collection

Data collection took place individually, in a single session lasting approximately two hours. Undergraduate and graduate students were previously trained to carry out the interview and data collection, from July 2019 to March 2020.

The variables of interest in this study were investigated using the following instruments:

- Sociodemographic characterization of the elderly: gender (female; male), age (in years); marital status (married/have a partner; single; divorced/separated/divorced; widowed), retirement (yes; no), schooling (in years); religion (Catholic; Evangelical; Christian Congregation; Adventist; Spiritism; Protestant; Buddhist; Umbanda; don’t have; didn’t answer; other), practicing religion (yes; no), consider their income to be sufficient to obtain the items they need on a daily basis (yes; no), individual income (in reais. Minimum wage in 2019 R$998.00 and in 2020 R$1,045.00), family income (in Brazilian reais), number of people living in the house, number of living children, health insurance (yes; no).

- Family functionality: assessed using the Family APGAR instrument, which consists of five items that assess family dynamics: adaptation, companionship, development, affectivity and resolving capacity. The answers to each question range from 0 to 5 points. The final score is the sum of all the questions in each dimension and can be classified as: high family dysfunction - 0 to 8; moderate family dysfunction - 9 to 12 or good functionality – 13 to 20 points^(17)^.

- Social support: assessed by the Medical Outcomes Study (MOS) Social Support Scale: made up of 19 questions, assigned to five dimensions of social support: material, affective, positive social interaction, emotional, informational. The answer obtained for each dimension is acquired by the frequency that the individual considers available for each type of support. Thus, never (0), rarely (1), sometimes (2), almost always (3) and always (4). The final score varies between 0 and 100 points, and the higher the score, the higher the level of perceived support^(18)^.

### Data Analysis and Processing

In order to characterize the profile of the sample, descriptive and comparative statistical analyses were carried out. The data was presented in tables, with absolute (n) and relative (%) frequencies calculated for categorical variables and measures of position and dispersion (mean, standard deviation) for continuous variables. The Kolmogorov-Smirnov test was applied to check that the variables adhered to a normal distribution, identifying the absence of normality. The Mann-Whitney, Chi-square and Fisher’s exact tests were used for comparison analyses. The level of significance adopted was (p < 0.05).

### Ethical Aspects

The invitation to take part in the study was sent to all the elderly people who met the aforementioned inclusion criteria. For those who accepted, a new home visit was scheduled and, prior to the assessment, the Informed Consent Form (ICF) was signed. The entire research process took place in accordance with the Research Ethics Committee of the Federal University of São Carlos (opinion no. 3.275.704, 22/04/2019). All the ethical precepts contained in Resolution 466/12 of the National Health Council were respected.

## RESULTS

The study sample consisted of 123 elderly people, who were divided into two groups according to family functionality: good family functionality (67.48%) and family dysfunction - moderate or severe (32.52%).

Table 1 shows the descriptive and comparative analyses of the demographic characteristics of the elderly according to family functionality.

**Table 1:** Descriptive and comparative analyses of the demographic characteristics of elderly people in a context of high social vulnerability according to family functionality. São Carlos, SP, Brazil, 2019-2020.

Variables	Total (n = 123)	Good functionality (n = 83)	Moderate/severe dysfunctionality (n = 40)	*p*-value
**Sex – n (%)**				0.489*
Women	67 (54.47%)	47 (56.63%)	20 (50.00%)	
Men	56 (45.53%)	36 (43.37%)	20 (50.00%)	

**Age (years) – Mean (SD)**	69.88 (6.91)	69.41 (6.39)	70.85 (7.89)	0.391†

**Marital Status – n (%)**				0.059‡
Married/Has a partner	114 (92.69%)	80 (96.40%)	34 (85.00%)	
Single	1 (0.81%)	1 (1.20%)	0 (0.00%)	
Divorced/Separated/Unmarried	4 (3.25%)	1(1.20%)	3 (7.50%)	
Widowed	4 (3.25%)	1 (1.20%)	3 (7.50%)	

**Retired/Pensioner – n (%)**				0.371*
Yes	98 (79.67%)	68 (81.93%)	30 (75.00%)	
No	25 (20.33%)	15 (18.07%)	10 (25.00%)	

**Schooling (years) – Mean (SD)**	3.03 (2.92)	3.02 (2.88)	3.05 (3.02)	0.936†

**Religion – n (%)**				0.323‡
Catholic	66 (53.66%)	49 (59.04%)	17 (42.50%)	
Evangelical	40 (32.52%)	24 (28.92%)	16 (40.00%)	
Christian Congregation	11 (8.94%)	7 (8.43%)	4 (10.00%)	
Spiritism	1 (0.81%)	1 (1.20%)	0 (0.00%)	
None	5 (4.07%)	2 (2.41%)	3 (7.50%)	

**Religious activities practice – n (%)**				0.684*
No	31 (25.20%)	20 (24.10%)	11 (27.50%)	
Yes	92 (74.80%)	63 (75.90%)	29 (72.50%)	

**Enough income – n (%)**			0.972*
No	71 (57.72%)	48 (57.83%)	23 (57.50%)	
Yes	52 (42.28%)	35 (42.17%)	17 (42.50%)	

**Individual income / Elderly (Brazilian Reais) – Mean (SD)**	1201.87 (866.49)	1218.57 (884.69)	1163.36 (833.91)	0,657†

**Family income (Brazilian Reais) – Mean (SD)**	2328.39 (1121.93)	2332.08 (1080.77)	2321.10 (1213.71)	0.907†

**Number of persons in the household – Mean (SD)**	2.85 (1.33)	2,96 (1,46)	2.63 (0.97)	0.276†

**Number of surviving descendants – Mean (SD)**	3.86 (2.23)	3.80 (2.22)	4.00 (2.27)	0.616†

**Health Insurance Plan – n (%)**				0.509*
No	108 (87.80%)	74 (89.16%)	34 (85.00%)	
Yes	15 (12.20%)	9 (10.84%)	6 (15.00%)	

Source: Research data, 2020. SD = Standard Deviation; *Chi-square; †Mann-Whitney; ‡Fisher’s Exact. The minimum wage in 2019 was R$998.00 and in 2020, R$1,045.00.

There was no noticeable statistically significant difference between the good family functionality and family dysfunction groups in terms of sociodemographic characteristics. It is worth noting that, due to the high level of social vulnerability and the inclusion criteria relating to the age of the sample, the elderly people interviewed had little schooling and low individual and family income. The average time spent studying was 3 years, of which 24.4% (n = 30) had never attended formal school. In addition, in minimum wages (MW), the individual income of the elderly was 1.15 MW and the family income was 2.23 MW – the significant participation of the elderly in the family budget (51.57%) in a context of vulnerability was evidenced in the data presented.

Table 2 shows the comparison between family functionality and social support for the elderly.

**Table 2:** Comparative analysis of the social support variable of elderly people in a context of severe social vulnerability according to family functionality. São Carlos, SP, Brazil, 2019-2020.

Social support	Total (n = 123)	Good functionality (n = 83)	Moderate/severe dysfunctionality (n = 40)	*p*-value*
**Material support – Median (SD)**	90.00 (18.12)	95.00 (16.03)	87.50 (20.73)	**0.009**
**Affective support – Median (SD)**	100.00 (18.33)	100.00 (17.15)	86.67 (19.85)	**0.008**
**Emotional support – Median (SD)**	85.00 (22.53)	90.00 (20.56)	65.00 (23.30)	**<0.001**
**Positive social interaction – Median (SD)**	80.00 (21.89)	85.00 (18.64)	65.00 (24.97)	**0.002**
**Information support – Median (SD)**	85.00 (22.70)	90.00 (20.04)	70.00 (24.12)	**<0.001**

Source: Research data, 2020. SD = Standard Deviation; *Mann-Whitney.

Regarding the perceived social support, the highest score was obtained in the affective support dimension and the lowest in positive social interaction. There was a statistically significant difference between the total score and all the dimensions of social support according to the family functionality groups. The group with good family functionality obtained higher median social support scores: affective support 100.00; material support 95.00; information support 90.00; emotional support 90.00; positive social interaction 85.00; when compared to the group with moderate or severe family dysfunction: affective support 86.67; material support 87.50; information support 70.00; emotional support 65.00; positive social interaction 65.00.

**Figure 1:**
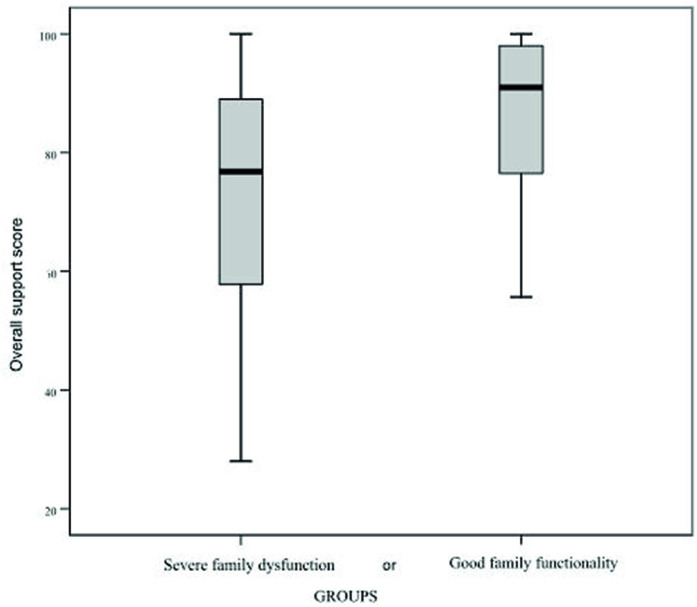
Comparison of the overall social support score of elderly people in a context of high social vulnerability according to family ­functionality. São Carlos, SP, 2019-2020.

Figure 1 shows the overall social support score according to the family functionality groups.

## DISCUSSION

The study compared the social support as perceived by elderlies according to family functionality. There was a significant difference in the overall score and all the dimensions of social support according to family functionality. Thus, elderly people living in environments with family dysfunction had lower median social support scores compared to those living in an environment with good family functionality.

The groups were similar in terms of sociodemographic characteristics, with a prevalence of women, married, with low levels of education, Catholic and retired. The findings corroborate the literature on the profile of the elderly in a context of high social vulnerability^(12,19)^. Schooling and economic conditions are significant determinants and protective factors for self-care, as people with higher levels of income and schooling have better access to information that contributes to healthy lifestyle habits, as well as having a higher level of social participation and family functionality, while those with low educational and economic levels have more affective and emotional disorders^(19,20)^.

With regard to family functionality, 67.48% of the elderly had good family functionality, which refers to the ability to adapt and maintain affective relationships and the members’ ability to solve problems^(11)^. This finding is similar to what is found in the Brazilian literature^(21,22)^. The family plays an important role in society, such as providing support, affection and protection, especially for the elderly. In this way, good family functionality helps to promote the physical and mental health^(11)^ and quality of life of the elderly^(21)^.

A study carried out in China with 1,186 elderly people found that participants with family dysfunction experienced more negative emotions when compared to elderly people with good functionality. In addition, elderly family members with dysfunction were more likely to develop anxious and depressive symptoms^(23)^.

In this study there was a statistically significant difference in the total score and all the dimensions of social support according to family functionality, i.e., elderly people belonging to environments with family dysfunction had lower social support scores compared to those in an environment with good family functionality. There was a higher average score for affective support, both for the group with good family functioning and for the group with family dysfunction. Similarly, a recent study also found that affective support was the most important support perceived by elderly people, reinforcing how affection is a determining factor in feelings of support and in building functional social bonds^(24)^.

The literature indicates that family dysfunction affects social support in older people^(11,22,25)^. In addition, lower social engagement, the absence of support networks and low socioeconomic status predict functional and cognitive decline, as well as a higher occurrence of chronic diseases^(5,26,27)^. A study of 2,052 elderly Brazilians showed that poor cognitive function, greater dependence and not having children were predictors of low family function. However, living with someone else, rather than alone, was considered an important predictor of adequate family function. The authors indicated that in old age, lack of autonomy, dependence, dementia and lack of social support affect the quality of life of the elderly. It is believed that the presence of family members increases the safety of the elderly, as they can help with daily activities and also contribute to social development^(28)^. It is worth noting that one of the inclusion criteria for this study was living with another elderly person, which may have influenced the results obtained in relation to family functionality and social support.

Elderly people’s participation in environments that promote social interaction and the acquisition of new knowledge can have repercussions on their emotional and psychological conditions, which have an impact on their perception of family relationships^(11)^. However, it is well known that elderly people who attend Open Universities for the Third Age have better health conditions, higher educational levels and different socio-demographic and economic characteristics than people who are socially vulnerable^(29)^.

In short, the findings of this study have potential implications for the development of social policies or recommendations to strengthen support networks in old age. Health professionals should encourage the formation of support networks that can meet the needs of older people, especially those living in environments with family dysfunction. The results of this study should be viewed with caution, as it has a cross-sectional design in a convenience sample, so there are limitations in terms of generalization and inferring causality. In addition, the IPVS was proposed by SEADE in 2010^(16)^, at which time the prevailing minimum wage was R$510.00. During the data collection period, the value of the minimum wage was between R$998.00 and R$1,045.00 in 2019 and 2020. Although the minimum wage was adjusted from 1994 to 2019, retirees and pensioners, who are the majority of the participants in this study (79.67%) accumulated historical wage losses of 87.28%. In 2019, when data collection began, the readjustment for pensioners earning above the minimum wage was set at 3.43% - this rate was lower than the readjustment of the minimum wage set at 4.61%^(30)^. This data shows that elderly people who were highly socially vulnerable in 2010 are more vulnerable to a shortage of resources in 2019 and 2020.

## CONCLUSION

There was a statistically significant difference in the total scores and in all the dimensions of social support of elderly people distributed according to family functionality. The group with good family functionality had higher social support scores when compared to the group with moderate or severe family dysfunction. In view of the above, it is suggested that longitudinal studies be carried out in order to understand how these variables behave over time in relation to elderly people. In addition, comparative research carried out with elderly people in different contexts of social vulnerability could also be useful.
